# Antennal transcriptome profiles of anopheline mosquitoes reveal human host olfactory specialization in *Anopheles gambiae*

**DOI:** 10.1186/1471-2164-14-749

**Published:** 2013-11-01

**Authors:** David C Rinker, Xiaofan Zhou, Ronald Jason Pitts, Antonis Rokas, Laurence J Zwiebel

**Affiliations:** 1 Center for Human Genetics Research, Vanderbilt University Medical Center, Nashville, Tennessee, USA; 2 Department of Biological Sciences, Vanderbilt University, Nashville, Tennessee, USA; 3 Department of Pharmacology, Vanderbilt Brain Institute, Program in Developmental Biology and Institutes of Chemical Biology and Global Health, Vanderbilt University Medical Center, Nashville, Tennessee, USA

**Keywords:** Anopheles, Mosquito, Antenna, Transcriptome, Olfaction, Malaria, Host-seeking, Odorant receptor, Molecular evolution, RNAseq

## Abstract

**Background:**

Two sibling members of the *Anopheles gambiae* species complex display notable differences in female blood meal preferences. *An. gambiae s.s.* has a well-documented preference for feeding upon human hosts, whereas *An. quadriannulatus* feeds on vertebrate/mammalian hosts, with only opportunistic feeding upon humans. Because mosquito host-seeking behaviors are largely driven by the sensory modality of olfaction, we hypothesized that hallmarks of these divergent host seeking phenotypes will be in evidence within the transcriptome profiles of the antennae, the mosquito’s principal chemosensory appendage.

**Results:**

To test this hypothesis, we have sequenced antennal mRNA of non-bloodfed females from each species and observed a number of distinct quantitative and qualitative differences in their chemosensory gene repertoires. In both species, these gene families show higher rates of sequence polymorphisms than the overall rates in their respective transcriptomes, with potentially important divergences between the two species. Moreover, quantitative differences in odorant receptor transcript abundances have been used to model potential distinctions in volatile odor receptivity between the two sibling species of anophelines.

**Conclusion:**

This analysis suggests that the anthropophagic behavior of *An. gambiae s.s*. reflects the differential distribution of olfactory receptors in the antenna, likely resulting from a co-option and refinement of molecular components common to both species. This study improves our understanding of the molecular evolution of chemoreceptors in closely related anophelines and suggests possible mechanisms that underlie the behavioral distinctions in host seeking that, in part, account for the differential vectorial capacity of these mosquitoes.

## Background

*Anopheles gambiae sensu stricto* is the major sub-Saharan vector for the human malaria parasite *Plasmodium falciparum* and the nominotypical member of a set of morphologically indistinguishable species that comprise the *Anopheles gambiae* complex [1]. The two molecular forms of *An. gambiae* s.s. (M and S), along with *Anopheles arabiensis*, constitute the major malaria vectors within this species complex. Despite their close evolutionary relationship, other members of the complex display either little (*Anopheles merus, Anopheles melas* and *Anopheles bwambae*) or no (*Anopheles quadriannulatus* A and *Anopheles quadriannulatus* B) vectorial capacity for human malaria [2].

Interestingly, the sole non-vector member of this species complex, *An. quadriannulatus* nevertheless is competent for *P. falciparum* infection [3,4] and molecular evidence suggests that the karyotype for this species derived directly from that of the main vector *An. gambiae* s.s. [5]. However, *An. quadriannulatus* is still considered to be a non-vector because its zoophagic [6,7], or at least highly opportunistic [8], host-preference effectively disrupts the human-to-human cycle of transmission required by *P. falciparum.* In contrast, female *An. gambiae* s.s. are especially efficient at transmitting human diseases because they preferentially obtain blood meals from human hosts, a behavioral trait (anthrophagy) of relatively recent origin [9,10].

Host seeking in mosquitoes is strongly influenced by olfactory and other sensory cues transduced by a variety of proteins that comprise the relevant transduction pathways [11,12]. In mosquitoes, olfactory genes are expressed in and around olfactory receptor neurons (ORNs) that are themselves contained within specialized chemosensory tissues and structures [13-18]. In most arthropods, ORNs are most highly concentrated within the antenna and, in mosquitoes, modulation of antennal ORN physiology has been correlated with some behavioral phenotypes [19,20]. The expression patterns of these genes along with the heterologous deorphanization of odor sensitivities of the sensory receptors that are central to these processes have helped refine our understanding of the links between chemosensory driven signaling and behavior [13,15,21-26]. Therefore, there is reason to suspect that species-specific, phenotypic variation between olfactory mediated behaviors may be informed by examining variation displayed by chemosensory genes, in terms of both molecular sequence and transcript abundance [27-30].

Several chemosensory gene families have been identified in *An. gambiae*, including odorant (*Agam\Ors*, hereafter referred to as *AgOrs*), gustatory (*Agam\Grs*, hereafter referred to as *AgGrs*), and variant ionotropic glutamate (*Agam\Irs*, hereafter referred to as *AgIrs*) receptors, as well as odorant binding proteins (*Agam\Obps* hereafter referred to as *AgObps*) [22,31-33]. These large multigene families encode proteins that are likely to account for the majority of chemical sensitivities in adult peripheral sensory appendages. For example, most *AgOrs* are transcribed in the antennae [21,27] and transcript abundances of many *AgOrs* are altered following a bloodmeal [28]. Furthermore, examination of AgOr response profiles in heterologous expression assays has identified numerous compounds from diverse chemical classes that are known activators of ORNs and behavioral attraction [25,26,34]. For example, among the recognized AgOr ligands are components of human sweat that have been implicated in *An. gambiae* host-seeking [35-38]. These alterations in *AgOr* transcript abundance in response to specific cues lead to apparent shifts in the potential receptivity of female antennae, including an enhancement of the response to 2-propylphenol, a compound that can act as an oviposition stimulant [28].

Most *AgGrs*, like their *D. melanogaster* counterparts, are assumed to encode receptors for sweet and bitter compounds as well as for other tastants [22,39]. However, transcripts for a small number of *AgGrs* are also enhanced in adult antennae where they may function in volatile chemical reception [27]. Moreover, three of the *AgGrs* encode palp-expressed receptors for carbon dioxide, an important activator of upwind flight in female mosquitoes [7,23,40].

While the *AgIrs* have not been fully characterized, many members of this gene family are expressed in adult appendages [27]. Based on their functions in *An. gambiae* larvae [32] and homologies to *D. melanogaster DmIrs *[33,41], the *AgIrs* are potential receptors for amines and acids which comprise host kairomones whose ORN sensitivities are housed in the basiconic, or grooved peg, antennal sensilla [19]. Several *AgIrs* show reduced transcript abundance following a bloodmeal, leading some to hypothesize this chemoreceptor family may also contribute to the observed host-seeking refractoriness in recently-bloodfed females [28].

*AgObp* transcripts are broadly present in adult head appendages at very high levels [16,27,42]. In addition, transcripts for many *AgObps* are specifically enhanced in body tissues, where their function remains uncharacterized. Nonetheless, knockdowns of antennal-expressed *Obp1* in mosquitoes leads to impaired indole responsiveness and indicates that OBPs function in mosquito peripheral odor sensitivities [43,44].

To test whether phenotypic host preference may be associated with the peripheral expression profiles of chemosensory genes such as chemoreceptors and *Obps*, we have isolated and sequenced mRNA from the antennae of *An. gambiae* and *An. quadriannulatus* to compare their transcriptome profiles in non-blood fed, female mosquitoes. We found that while there were high levels of similarity in the type and number of chemosensory genes detectable in the antennae of both species, there were significant divergences at both the molecular and transcriptional levels. Furthermore, differences in the antennal chemoreceptor composition, most notably within the *OR* family, appeared to express a subset of the *An. quadriannulatus* chemosensory repertoire within *An. gambiae*, that may be particularly refined for the detection of human associated host cues.

## Results and discussion

### *An. gambiae* and *An. quadriannulatus* share highly similar chemosensory gene repertoires

**Figure 1:**
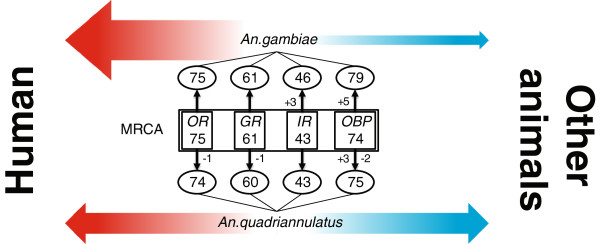
**Chemosensory gene repertoires of *An. gambiae *and *An. quadriannulatus*.** The numbers of *An. gambiae* and *An. quadriannulatus* chemosensory genes annotated in this study (ovals). The estimated numbers of chemosensory genes in the most recent common ancestor (MRCA) of the two species (boxes). The numbers along vertical arrows indicate the estimated numbers of gene gain (+) and loss (-) events. The red and blue schematic arrows indicate the host preferences (**red**: anthropophagic, **blue**: zoophagic) of *An. gambiae* and *An. quadriannulatus*.

We first compared the number of chemosensory genes in the genomes of *An. gambiae* and *An. quadriannulatus*. While the size and composition of *An. gambiae* chemosensory gene families have been reported previously [22,27,31], little is known about *An. quadriannulatus* since its genome sequence has only recently become publically available (https://olive.broadinstitute.org/projects/anopheles). To fully characterize the chemosensory repertoire in *An. quadriannulatus*, we conducted exhaustive and iterative searches for homologs of known insect chemosensory genes using a rigorous pipeline and carefully annotated gene models [45] (see Materials and Methods). The same procedure was also applied on *An. gambiae* to eliminate potential bias introduced by gene annotation. In total, we identified 74 *OR*s, 60 *GR*s, 43 *IR*s, and 75 *OBP*s in *An. quadriannulatus*, as well as 75 *OR*s, 61 *GR*s, 46 *IR*s, and 80 *OBP*s in *An. gambiae* (Figure 1).

Comparison of the *An. gambiae* and *An. quadriannulatus* annotations revealed the presence of a relatively stable overall number of chemosensory genes in the two species, which suggests that their repertoires are fairly conserved; although we cannot entirely rule out the hypothesis of rapid underlying gene turnover despite that the total gene number has remained unchanged. To distinguish between these two scenarios, we further investigated the evolutionary dynamics of chemosensory genes in *An. gambiae* and *An. quadriannulatus*. Based upon our phylogenetic analysis (Additional file 1: Dataset S1), chemosensory genes can be classified into 253 orthologous groups (OGs), including 75 OGs of *OR*s, 61 OGs of *GR*s, 43 OGs of *IR*s, and 74 OGs of *OBP*s (Figure 1), where each OG represents a single gene in the most recent common ancestor (MRCA) of the two species. In this view, the estimated number of chemosensory genes in the MRCA is nearly identical to the number we observe in the two present species (Figure 1). Furthermore, the vast majority of OGs are comprised of one gene from each species; only 12 of the 253 OGs show evidence for one or two gene gain or loss events (Figure 1). Taken together, these data support the hypothesis that the chemosensory gene repertoire has been stable following the speciation of *An. gambiae* and *An. quadriannulatus*.

Rapid gene birth-and-death is a signature feature of the molecular evolution of insect chemosensory genes [46], as revealed by comparisons of species with various levels of relatedness and varying reliance on chemosensation. Lineage-specific variations in the size of chemosensory families are usually correlated with altered requirements on chemosensation posed by changes in life style and ecology [47,48]. As shown in the comparative studies of generalist and specialist sister species in *Drosophila*, *D. sechellia* likely underwent dramatic host specialization after its divergence from the generalist *D. simulans *[49-52]. This behavioral change was accompanied by both an accelerated rate of gene loss and an elevated level of sequence divergence of chemosensory receptors of *D. sechellia*, likely reflecting a reduced, but more focused chemosensory capability due to the more restricted host range associated with geographic isolation [49-52]*.*

In contrast, our comparison of the four chemosensory families between the anthropophilic mosquito *An. gambiae* and its zoophilic sibling *An. quadriannulatus*, revealed only slight variations in gene number. Indeed, the two species differ by only one gene in both *OR* and *GR* families although these two types of receptors are vital for mosquito host seeking and preference. Among other chemosensory genes, the three *An. gambiae* specific *IR* duplicates belong to the subfamily of “divergent IRs” which are most abundant outside antennae [27,32,33]; there is a paucity of functional information for the *OBPs* that have been duplicated or lost, thus a rationale for these events remains elusive.

This discrepancy in the evolutionary pattern could be due to several factors. Firstly, the divergence of *An. gambiae* and *An. quadriannulatus* is estimated to have occurred very recently, as little as only several thousand years ago [10,53], coinciding with the increased availability of human hosts that paralleled the growth of agriculturally-based communities; this is significantly less than the ~0.5 million years separation of *D. sechellia* and *D. simulans *[54]. Our results suggest that different modes of chemosensory gene evolution have played major roles at different time-scales; genomic changes at levels other than gene copy number are likely to have rapidly driven the behavioral divergence between the two mosquitoes over a very short period of time. Moreover, the zoophagy of *An. quadriannulatus* likely represents the ancestral state and *An. gambiae* acquired the preference for humans later [9]. It has been suggested for phytophagous insects that the specialization to a fraction of its ancestral host range usually involves altered sensitivity to odors for both previous and new hosts (for preference) [55]. Similarly, the adaptation of *An. gambiae* to human hosts may have required more acute responses to both attractants of human origin and deterrents of animal origin in comparison to *An. quadriannulatus*. Such differences could have been achieved either by functional divergence or by differential expression/abundance of orthologous chemosensory genes between the two mosquitoes, or both.

### Chemosensory genes underwent rapid sequence evolution

Our overall comparison of chemosensory genes between *An. gambiae* and *An. quadriannulatus* raises the possibility that, given the largely shared repertoire, the functional divergence between orthologs may be an important factor underlying the shift in host preference. That said, the lack of any structural insight and functional data for most chemosensory genes hinders a direct comparison of ligand sensitivities between orthologous genes. However, the role of functional divergence can still be assessed in part by examining the pattern of chemosensory gene evolution at the sequence level. To begin to address this, we investigated the evolution of each of the 241 one-to-one orthologous pairs of chemosensory genes by using two metrics: (1) the rate of amino acid substitution (protein distance), which represents the rate of protein sequence divergence; and (2) the ratio of non-synonymous substitution rate to synonymous substitution rate (dN/dS ratio), which estimates the influence of natural selection on protein coding sequences (Additional file 2, Table S1).

**Figure 2:**
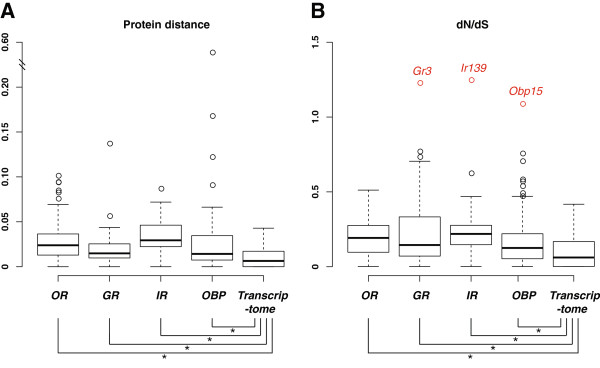
**Evolutionary rates of anopheline chemosensory genes.** Box plots of **(A)** protein distances and **(B)** dN/dS ratios between orthologous pairs of *OR*, *GR*, *IR*, and *OBP* genes. Orthologous gene pairs delineated from *An. gambiae* genome and *An. quadriannulatus* transcriptome assembly were used as control. Wilcoxon rank sum tests showed that evolutionary rates were significantly different between chemosensory gene families and the control (* denotes *p*-value < 0.001). Outliers (circles) are shown for chemosensory gene families but not for the transcriptome background due to their large numbers. Ortholog pairs having a dN/dS value greater than one are noted in **red**.

As shown in Figure 2, while there are considerable variations in evolutionary rates among chemosensory genes, all four chemosensory families have significantly higher median values of protein distance and dN/dS ratio as compared to other genes, suggesting that chemosensory genes as a whole evolved more rapidly than their respective transcriptome backgrounds. Among gene families, the *IR*s display the highest median values of both measurements, mostly driven by the “divergent *IR*s”, followed by *OR*s and *GR*s. While *OBP*s appear to have somewhat overall lower evolutionary rates, some of the most rapidly evolving chemosensory genes are also found in this family. Within each family, genes are broadly distributed across the range of protein distance and dN/dS ratios. While genes encoding OR and IR co-receptors and GR carbon dioxide receptors show extremely low evolutionary rates, there are 3 genes with dN/dS ratios > 1 (*Gr3*, *Ir139*, and *Obp15*), and a number of others with dN/dS ratios around 0.5. While large dN/dS ratios (> 1) are considered to be evidence for positive selection, intermediate values may indicate relaxed purifying selection, or they could reflect positive selection on a fraction of the gene sequence.

These two measurements of evolutionary rate show an overall positive correlation in all four chemosensory families (Additional file 3: Figure S1). However, there are also multiple examples where orthologous gene pairs display high dN/dS ratios but only a small number of amino acid changes (e.g. Or24, Or36, Gr3, Gr57, and Ir141). These genes are most likely the result of positive selection; while both positive selection and relaxed purifying selection can lead to elevated dN/dS ratios, genes under relaxed purifying selection would also be expected to have a higher rate of amino acid substitution than is seen here.

Genes under both types of selection represent potential candidates for genomic determinants of the behavioral and electrophysiological response differences between *An. gambiae* and *An. quadriannulatus*. Differential odor responses that are mediated by functional divergence of chemosensory genes would most likely require positive selection on genes that are responsible for the detection of human attractants and/or non-human deterrents, leading to increased sensitivity for these semiochemicals. On the other hand, receptors whose ligands include human deterrents and non-human attractants would possibly experience relaxed selective constraints as amino acid changes that attenuate these responses would be less deleterious or even beneficial. To look for additional evidence of functional divergence, we characterized the rate of conservative and radical amino acid substitutions and the distribution of these substitutions on the primary sequences of OR proteins. In contrast to conservative and typically neutral substitutions, radical amino acid substitutions are more likely to alter protein function; therefore the ratio of radical substitution rate to conservative substitution rate (dR/dC) is also a very useful measurement of selective pressure on protein evolution [56,57]. Using this metric, we identified dR/dC ratios > 1 for 6 *Or*s, 12 *Gr*s, 4 *Ir*s, and 3 *Obp*s (Additional file 2: Table S1), suggesting these genes might also be under positive selection.

Insect *OR* genes encode 7 transmembrane (TM) proteins and at least one previous study has suggested that TM domains in OR proteins participate in receptor-ligand interaction [58]. On that basis, we performed topology predictions for all *Ag/AqOrs* and counted conservative and radical substitutions specifically within the predicted TM domain regions. In total, 56 out of 71 ORs have one or more amino acid substitutions in TM domains, including at least one radical substitution in 43 OR genes (Additional file 4: Figure S2 and Additional file 5: Table S2). Inasmuch as negatively charged amino acids such as glutamic acid, asparagine, and tyrosine are involved in defining OR function [59], the frequency of replacements targeting these specific residues was also assessed. Indeed, 38 out of 71 OR proteins contain at least one substitution of a negatively charged residue and 6 ORs manifest these substitutions within predicted TM domains. Taken together, our comprehensive sequence analyses of chemosensory genes have identified multiple types of alterations that suggest that some degree of functional divergence may have occurred between these closely related sibling species of anophelines.

### Chemosensory genes are differentially abundant between the two species

To address the contribution of changes in chemosensory gene transcript levels to the behavioral differences between *An. gambiae* and *An. quadriannulatus*, we compared the antennal transcriptome profiles of the two species, focusing specifically on the differential enrichment of chemosensory genes in each of the *OBP*, *IR*, and *OR* families (the *GR* family was not discussed here due to the lack of meaningful antennal expression). Among the transcripts detected in the antenna of *An*. *quadriannulatus*, our findings were broadly consistent with previous RNAseq studies in *An*. *gambiae *[27,28], and both species showed extensive conservation in the number and identity of detectable, chemosensory genes (Additional file 6: Table S3). Indeed, we only identified a few instances of species-specific chemoreceptor expression; the most notable occurrences were *Or33* in *An*. *quadriannulatus* and *Ir7s* in *An*. *gambiae* which both displayed transcript abundance levels above the median level for all transcripts. Such profound overlap in the variety of expressed, chemosensory genes may not be surprising given the level of genomic conservation and serves to reinforce the evolutionary proximity of these two species.

**Figure 3:**
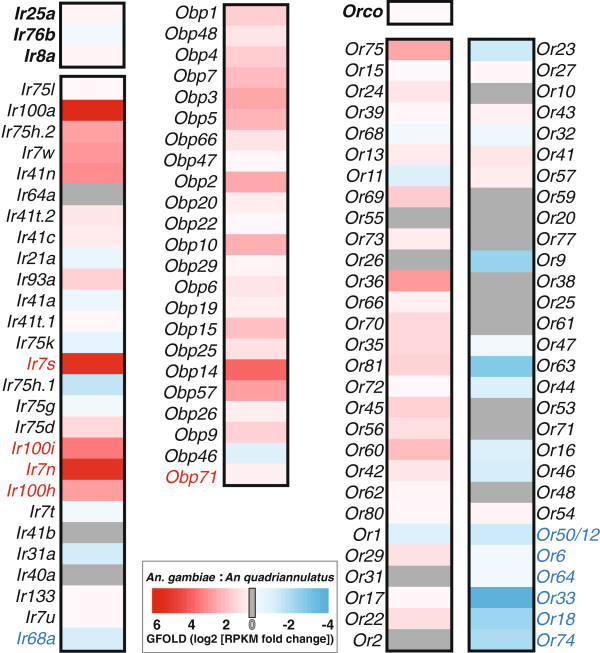
**Differential antennal abundances of chemosensory genes.** Heat maps for the three most abundant chemoreceptor gene families in the antenna. Only transcripts that were detectable one or both species are displayed. Colors indicate a normalized, GFOLD score (reliable log2 fold difference in transcript abundance) denoting enrichment in either *An. gambiae* (**red**) or *An. quadriannulatus* (**blue**); genes showing no discernible expression differences between the species (GFOLD=0) are shown in grey. The gene names are ordered (from high to low, top to bottom) based upon their relative abundance in *An. gambiae.* Co-receptors are displayed in bold above the *OR* and *IR* gene family heat maps. Chemoreceptor names are color coded if they were only classified as being detectable in *An. gambiae* (red) or in *An. quadriannulatus* (blue).

The most abundant chemosensory gene family in the mosquito antenna was the *OBPs*. The antennal *OBPs* in both *An. gambiae* and *An. quadriannulatus* belonged exclusively to the “classical” subclass of *OBP*, an observation consistent with our previous study that detected “atypical” *AgObps* in the antennae at only a single, discreet time point following a bloodmeal [28]. All *OBP* transcripts were much more abundant in the antennae of *An*. *gambiae*, with the total RPKM of detectable *OBPs* nearly twice that for the *OBPs* found in *An*. *quadriannulatus*. Indeed, the *OBPs* were the only family of chemosensory genes that was overrepresented in *An. gambiae*, with every detectable *OBP* displaying a significant difference in transcript abundance (Figure 3). Interestingly, despite the nearly 2:1 disparity in gross, *OBP* transcript abundance, the expression-based rank order of *OBPs* remained highly conserved between the two species (Spearman’s r=0.94), and was more highly correlated than that of either the *IRs* (r=0.70) or the *ORs* (r=0.64). While on the whole, the *OBP* gene family plays diverse roles in insects, the similar presence and distribution of this distinct subset of *OBPs* between *An. gambiae* and *An. quadriannulatus* suggests they are more conserved within anopheline olfactory tissues, relative to the evolutionarily labile membrane bound, ligand specific chemoreceptors.

In contrast to the *OBPs*, the *IRs* and *ORs* exhibited widespread variation in transcript abundances between *An. gambiae* and *An. quadriannulatus* (Figure 3). The antennal *IRs* displayed the most instances of transcript variation, with 27 of the 30 detectable *IRs* showing significant differences in abundance. Moreover, the sum total of the presumptive *IR* co-receptors (i.e., the combined RPKM values for *Ir25a*, *Ir76b* and *Ir8a*) is more than 60% higher in *An. quadriannulatus.* This divergence in *IR* expression levels, in conjunction with the low coefficient of determination in the rank order of *IRs* between *An. gambiae* and *An. quadriannulatus* (r^2^=0.49), distinguishes the *IRs* as the most variable chemosensory gene family between the sibling species. This greater variability parallels the molecular evolutionary analysis above, which showed the *IR* family to display the highest degree of sequence divergence among the chemo-receptors (Figure 2). Because the ultimate roles and functions of the individual members of *IR* family are still being defined, the precise impact of these observed differences is as yet unclear. However, the pervasive, interspecific variation in both sequence composition and transcript abundance may indicate that *IRs* are especially adaptable. This represents a chemoreceptor class that may be involved in both mediating internal signals as well as sensing external environmental cues.

The *ORs* are the best characterized class of chemoreceptor in insects. Many functional aspects of dipteran *ORs* have been determined for both *D. melanogaster* and *An. gambiae*, and the results consistently show that individual *Dm- and AgOrs* display their own distinct range of odor selectivity or “tuning” [25,26]. For example a recent report suggests that *ORs* as a class are critical to defining mosquito host-specificity [60].

*ORs* are localized in the dendritic membranes of ORNs and require the presence of a conserved odorant receptor co-receptor (*Orco*) for correct localization and subsequent function [17,61]. Because *Orco* is always required for *OR* function, its abundance may be taken as a general proxy for overall *OR* abundance. By that measure, *An. gambiae* antennae displayed only a modest (6%) enrichment in the *Orco* transcript abundance compared with *An. quadriannulatus*, and we can reasonably conclude that the overall expression levels of *ORs* are consistent between the two species. Indeed, this conservation is in keeping with previous, comparative morphological studies that reported a slightly higher density of sensilla on *An. gambiae* antenna, including the highly abundant trichoid sensilla [62] that house three *Orco*-expressing ORNs [13,16]. Therefore, while *An. gambiae* antennae might possess a very slight advantage in *OR*-mediated odor sensitivity, our transcriptional data largely agrees with the comparative morphologic study to imply that that both species share equivalent olfactory capabilities [62].

Similarly, in both species half of the sum totals of tuning *OR* transcripts in the antenna were comprised of a small, largely identical subset of either 7 *ORs* in *An. gambiae* or 8 *ORs* in *An. quadriannulatus.* Within this top 50%, 5 *ORs* were shared between species (*Ors 11*, *15, 24, 68* and *75*) and had an average dN/dS below that of the *OR* class as a whole. Therefore, in terms of relative transcript abundance, most of the predominant antennal *Ors* shared between the species were also more conserved at the sequence level.

Beyond these similarities, the composition of the remainder of the tuning *OR* pool appeared to vary substantially between the two species (Figure 3). In total, 49 of 58 (84%) tuning *ORs* showed significant differences, 16 of which were more than a 2-fold enriched in one of the species.

**Figure 4:**
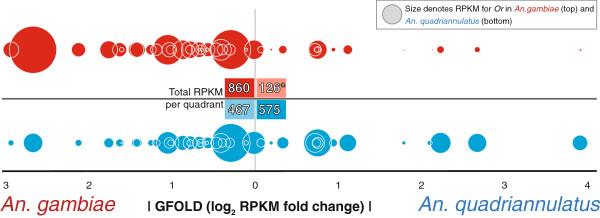
**Distribution of differentially abundant antennal *Ors *and their relative abundance levels in *An. gambiae *and *An. quadriannulatus*.** Individual tuning *Or* orthologs are represented by bubbles with areas scaled to their respective abundance (RPKM) in either *An. gambiae* (**red**) or *An. quadriannulatus* (**blue**). *Or* orthologs are arranged horizontally based upon their enrichment (GFOLD value) in either *An. gambiae* (left) or *An. quadriannulatus* (right). Total RPKMs for each quadrant are indicated in the center. The asterisk denotes the larger than expected proportion of *Or* abundance in *An. gambiae* ascribable to *Ors* that are also enriched in *An. gambiae* (Fisher’s Exact Test, *p*=2.2x10^-16^).

In *An. gambiae* antennae, the most noticeable overall trend in differential *OR* abundance was the degree to which select *ORs* were enriched as compared to *An. quadriannulatus* (Figure 4). While there were no *ORs* whose antennal expression appeared specific to *An. gambiae,* 29 tuning *ORs* showed significant levels of enrichment in *An. gambiae*, with *ORs 36*, *60*, *69*, and *75* each showing as much as a 4–6 fold enrichment (Figure 3). Overall, these *An*. *gambiae* enriched *ORs* were 6-fold more abundant than the combined pool of depleted *ORs*. This stands in marked contrast to the balanced distribution of *ORs* in *An. quadriannulatus*, with enriched and depleted *ORs* showing similar expression levels in terms of overall RPKM (Figure 4). Taken together, the *OR*-mediated odor coding of the *An*. *gambiae* antennae appears to be an overrepresented subset (Fisher’s Exact test, *p*=2.2x10^-16^) of *ORs* whose orthologs are also present in *An*. *quadriannulatus*. This sizeable skew in the distribution of *ORs* implies that the *An. gambiae* antenna predominantly expresses only a subset of those ORs within the antenna of *An. quadriannulatus.*

**Figure 5:**
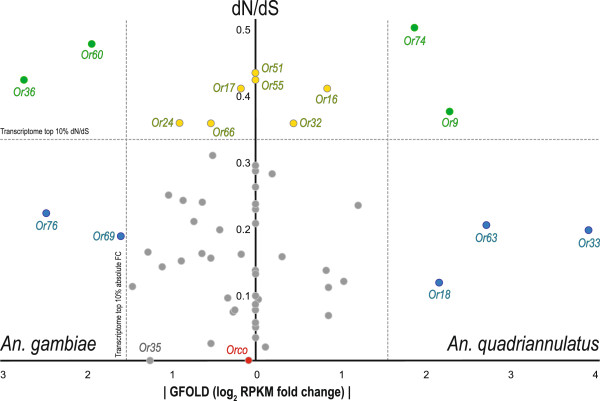
**Differential expression of antennal *ORs *plotted against dN/dS.***X-axis* represents the absolute GFOLD score (reliable log2 fold difference in transcript abundance) for *Ors* enriched in either *An. gambiae* (left half) or in *An. quadriannulatus* (right half). *Ors* displaying no significant difference in transcript abundance are plotted at zero. *Y-axis* is the interspecific dN/dS for each *Or*. *Ors* are color coded as follows: **grey:** conserved in sequence and in transcription, **blue:** conserved in sequence but diverged in transcription, **yellow:** diverged in sequence but conserved in transcription, **green:** diverged in sequence and in transcription. *Horizontal dashed line* denotes the top 10% of transcriptome wide dN/dS value and the *vertical dashed line* denotes the top 10% of transcriptome wide, absolute fold change.

When differential levels of *OR* transcripts were viewed within the context of molecular divergence (Figure 5), there was no significant correlation between transcript enrichment and dN/dS ratio. However, it was clear that *ORs* with higher evolutionary rates were also more variable in terms of transcript enrichment and tended to display higher enrichment levels. When *OR*s were analyzed in quartiles based on their dN/dS ratios, the upper three quartiles (dN/dS ratio ≥ 0.1) showed significantly higher median and variance values of transcript enrichment as compared with the first quartile, either individually or collectively (see Additional file 7: Table S4). Interestingly, the opposite trend was observed at the level of the antennal transcriptome profile, where genes in the first quartile (with lower dN/dS ratios) displayed greater magnitude and variability of transcript enrichment (see Additional file 7: Table S4). In addition, *OR*s with dN/dS ratios above the transcriptome median (0.0611) comprised the majority of detectable *OR*s and showed significantly higher levels of enrichment than those genes in the transcriptome background in the upper half of the dN/dS (Wilcoxon rank sum test, p=0.02792). This contrast, once again, highlights that *OR*s are under rapid evolution at both sequence and expression levels.

Overall, there were 11 and 9 *OR*s that resided in the top 10% of the transcriptome profile in terms of their evolutionary rates and absolute levels of transcript enrichment, respectively (Figure 5). Four of these *OR*s showed both high sequence divergence and abundance differences, while the remaining genes differed in either sequence or abundance. This pattern suggests that sequence divergence and differential abundance represent two non-mutually exclusive mechanisms for the evolution of *ORs*, and perhaps other chemosensory genes. Those *OR*s with exceptionally high levels of sequence divergence and/or transcript enrichment likely play important roles in chemosensory-mediated behavioral differences between *An. gambiae* and *An. quadriannulatus*. Some of the relatively more conserved *OR*s might be interesting as well. For instance, *Or35* is the most conserved tuning *OR* but its absolute fold change was ranked within the top 20% of the antennal transcriptome profile.

### Differential receptivity analysis

We have previously integrated *OR* functional data with RNAseq data to model the receptivity profile for the antenna of *An. gambiae* following a bloodmeal [28]. This analytical approach synthesized the effects of many small changes in the expression profiles of individual tuning *ORs* to treat the antenna as a single, chemosensory unit. Applying the same methodology here, to effectively map the *An. gambiae* odorant receptivity onto the *An. quadriannulatus* OR transcriptome profile, we modeled potential odor-coding differences between these two species*.* While it is important to note that this approach assumes the general functional conservation among interspecific *OR* orthologs, this is a reasonable assumption given that non-conservative substitutions observed among the *OR*s occur in the trans-membrane and intra-cellular loop regions and are therefore most likely to impact the channel properties of the Orco-OR complex rather than OR-ligand interactions [59].

**Figure 6:**
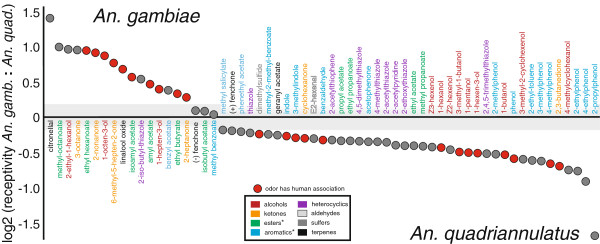
**Differences in *OR *mediated odorant receptivity between *An. gambiae *and *An. quadriannulatus *antennae.** Vertical axis represents computed, interspecific differences in antennal receptivity to a panel of odors. Displayed results are sorted left to right based upon the level of each odor’s relative receptivity enhancement in either *An. gambiae* (positive values) or *An. quadriannulatus* (negative values). The **grey** region around zero denotes an absolute change in relative receptivity of 10% or less. Chemical names are color coded by chemical class and asterisks denote chemical classes whose receptivity is disproportionately represented in one species (Fishers Exact Test, *p*<0.05). **Red** points denote odors that have been detected in human-associated skin emanations.

While the results of this analysis showed the species to share a similar level of receptivity toward three floral compounds (fenchone, isobutyl-acetate and methyl-benzoate), there appears to be a general reduction in relative receptivity within the *An. gambiae* antenna to many of the odorants tested. *An. quadriannulatus* appeared more receptive to a wide range of chemical classes including most aromatic compounds and many alcohols (Figure 6; Additional file 8: Table S5), and while many of these compounds are plant associated some are also components of human skin [36,38,63-65]. Of those compounds to which *An. quadriannulatus* appears more receptive, the two indolic compounds are known to be important to the chemical ecology of many mosquito species [36,37,64,66-68]. While both indole and 3-methylindole have been characterized as human associated compounds [36,64,69], they are also associated with other natural sources, including decaying organic material and animal excreta [66]. Accordingly, we cannot discount the possibility that the same odorant can elicit different perceptions dependent upon ecological context. Nevertheless, the presence of these compounds along with the several other human associated odorants can also be rationalized within the context of human host-seeking since *An. quadriannulatus* displays limited, anthropophagic behavior as well [70].

In contrast, the *OR*-mediated olfactory specialization of *An. gambiae* antenna appears to be heavily biased (Fishers Exact test, *p*=0.06) toward odors which have been previously associated with human skin emanation, including a majority of the esters assayed (Fishers Exact test, *p*=0.04). Furthermore, if we only consider compounds that showed a change in relative receptivity greater than 10% in either species that show only minimal, the apparent enhanced receptivity of *An. gambiae* to human-associated odor chemicals becomes even more significant (Fisher’s Exact test p=0.02). Moreover, some human associated odors have greater magnitudes of receptivity enhancement in *An. gambiae* to as compared to any of those in *An. quadriannulatus* (Figure 6). This notable trend agrees with both the molecular and the transcriptional analyses above, further suggesting that at the molecular level, the OR-mediated sensitivity of the antennae of *An. gambiae* appears to be more focused and specialized than that of *An. quadriannulatus.*

## Conclusions

In this study we examined the RNA composition of the peripheral chemosensory tissues of *An. gambiae s.s.* and *An. quadriannulatus*, two closely related members of the *An. gambiae* species complex. Because these two species are phenotypically divergent in terms of their host seeking predilections, we looked specifically at differences within the chemosensory gene classes, both at the molecular level and at the transcriptional level. Overall, while the chemosensory gene repertoire was highly conserved, we found that rates of evolution of each of the chemosensory gene families were more rapid than the genomic background. In particular, we identified considerable levels of radical amino acid changes between orthologous *OR* genes that may potentially result in functional differences. To our knowledge, this is the first comparative study of the chemosensory gene repertoire between sibling species that are diverged by only several thousand years ago. Unlike the dramatic copy number changes often observed in comparisons of more distantly related species, these results suggest that functional divergence between orthologous chemosensory genes may be key in driving behavioral differences in the immediate aftermath of speciation events.

A careful analysis of their antennal transcriptome profiles also revealed both the overall conservation of some critical chemosensory transcripts (e.g. Orco), along with large degrees of abundance differences among some individual gene family members. The observed similarities confirm results of prior morphological studies that reported the antennae of both species share similar sensilla densities overall [62]. Though no *ORs* appeared to be exclusively expressed within the *An. gambiae* antenna, the divergence in the overall transcriptional profile of the *ORs* was considerable. The specific *ORs* whose transcripts comprise the preponderance of *OR* transcripts within the antennae of *An. gambiae* are also greatly enriched as compared to *An. quadriannulatus*, indicating that in terms of *OR* composition, the *An. gambiae* antenna appears most likely to be a specialization of the *An. quadriannulatus* antenna*.*

When these interspecific abundance differences in the *OR* gene family members were integrated *in silico* with *AgOr* functional data, the resulting antennal “receptivities” again indicated that the human-biased odor receptivity of *An. gambiae* was most likely a refinement of that of *An. quadriannulatus*. Moreover, this biased receptivity of *An. gambiae* antenna toward human-derived odors may be further augmented by the functional differences between orthologous *ORs* suggested by our sequence analyses. Future functional tests of *AqOr* –odor tuning will further improve our understanding in this regard.

Taken together, and given the central role that *ORs* play in defining host specificity, the anthropophagy of *An. gambiae* is most likely not derived from the evolution of any single *OR* specific for the purpose of human host seeking. Instead, we posit the receptivity bias in the antenna of *An. gambiae* toward human host odors is likely the result of the cumulative effects of both functional divergences and changes in the abundance and distribution of common *ORs* already present within the *An. gambiae* species complex.

## Methods

### Gene annotation

The genome assemblies of *An. gambiae* (version AgamP3) and *An. quadriannulatus* (version 1) were downloaded from the websites of VectorBase (http://www.vectorbase.org) and Broad Institute (olive.broadinstitute.org), respectively. The annotation of chemosensory genes was performed following a previous protocol [45]. In brief, previously reported chemosensory genes from *An. gambiae*, *Aedes aegypti*, *Culex quinquefasciatus*, and *D. melanogaster* were used as queries in TBLASTN [71] searches against the two anopheline genomes. Putative chemosensory gene coding loci were identified after filtering out low-scoring blast hits. For each locus, the query sequence that yield the highest bit score was selected as reference to perform homology-based gene prediction using GeneWise (version 2.2.0; [72]). All gene models were manually inspected and modified if needed. All genomic data is available through VectorBase and the annotated chemoreceptor sequences are listed in supplementary Table S1.

### Phylogenetic analysis

For each of the *OR*/*GR*/*IR*/*OBP* families, protein sequences of genes in the two mosquitoes were aligned using MAFFT (version 7.037b; [73]). The multiple sequence alignments were manually curated and poorly aligned regions were removed using trimAl (version 1.4; [74]) with “automated1” option. Maximum-likelihood trees were constructed using RAxML (version 7.4.7; [75]) and the reliability of tree topology was evaluated with 100 bootstrap replicates. Resulting gene trees were reconciled with the species phylogeny to estimate ancestral gene copy numbers and gene gain and loss events. An orthologous group is defined as a highly supported clade (greater than 90%) representing a single gene in the common ancestor of *An. gambiae* and *An. quadriannulatus*.

### Analysis of sequence divergence

For each orthologous pair of chemosensory genes in *An. gambiae* and *An. quadriannulatus*, protein sequences were aligned using MAFFT and the corresponding nucleotide alignment was generated using a custom script (available upon request). The rate of amino acid substitution and dN/dS ratio were calculated using PROTDIST (from the Phylip package version 3.69) and CodeML (from the PAML package version 4.7; [76]), respectively. The dR/dC ratio was calculated using the Zhang method [77], for which radical and conservative amino acid changes were defined by the Dayhoff classes (“AGPST”, “DENQ”, “HKR”, “ILMV”, “FWY”, and “C”). The topologies of Or proteins were predicted using TOPCONS [78] and the number of radical/conservative amino acid changes in transmembrane domain regions were counted accordingly.

To identify additional orthologous gene pairs between the two mosquitoes, *de novo* transcriptome assembly of *An. quadriannulatus* was generated and likely coding regions were extracted, both using Trinity (version 2012-10-05; [79]) Orthologous groups were then constructed from annotated genes in *An. gambiae* (version AgamP3.7) and likely coding sequences in *An. quadriannulatus* using orthoMCL (version 2.0.5; [80]) Protein divergence, dN/dS ratio, and dR/dC ratio were calculated for each 1-to-1 orthologous pair similarly to chemosensory gene pairs.

### Mosquitos and mosquito rearing

*An. gambiae sensu stricto* (SUA 2La/2La, an M-form isolate originating from Suakoko, Liberia) and *An. quadriannulatus* (SKUQUA, an A form isolate originating from Skukuza, South Africa) were reared in the Vanderbilt Insectary Facility as described previously [21]. Adult mosquitoes were reared under 12:12 light–dark conditions and had constant access to 10% sucrose solution.

### RNA isolation and RNA sequencing

Four to six day old adult female mosquitoes from each species were collected in the middle of the light phase (~ZT6) for antennal resection. For each collection, antennae were hand-resected into TRIzol, and total RNA was isolated. mRNA isolation and cDNA library preparation were carried out using the Illumina mRNA sequencing kit (Illumina Inc.; San Diego, CA). Libraries were barcoded and sequenced in paired-end fashion (50PE *An. quadriannulatus*, 100PE for *An. gambiae*) on an Illumina HiSeq2000. Approximately 30 million reads were generated for each sample. No biological replicates were preformed becasue sample-to-sample variation in RNAseq results among anophelene antennae has been observed to be very low (Additional file 9: Figure S3).

### Data processing and abundance profiling

Individual Illumina read files (fastq) were trimmed and filtered using Trimmomatic, a software package specifically designed for trimming NGS reads. Paired end Trimmomatic parameters used were: LEADING:3 TRAILING:3 SLIDINGWINDOW:4:15 MINLEN:36. FastQC was used for data set quality checking.

To better quantify transcript abundances in *An. quadriannulatus*, a modified version of the *An. gambiae* reference genome was prepared to eliminate potential bias caused by genomic sequence differences between the two species. The reads of *An. quadriannulatus* were first mapped to the *An. gambiae* reference genome (version AgamP3) using Tophat2 (version 2.0.8) with the guidance of gene annotation (version AgamP3.7), and only one alignment was reported for each mapped read. Fixed differences between the species were called and filtered using SAMtools (version 0.1.18) with a minimum read depth of 5 and variant quality score of 60. We then replaced nucleotides in the *An. gambiae* reference genome at sites of fixed differences with each site’s most frequent, alternative allele. This modified reference genome sequence was used for subsequent analyses of *An. quadriannulatus* transcriptome. Finally, reads were then aligned to the respective, indexed genome using Tophat2 [81].

### Differential transcript abundance calculation

Statistical significance along with fold change was determined by pairwise comparison of the Tophat2 alignments for each of the two species using GFOLD (version 1.0.9 [82]) configured for a 99 percent confidence interval. The result was a set of GFOLD values (a.k.a. GFOLD’s “reliable” log2 fold change) for each *An. gambiae* gene identifier (AGAP); GFOLD values other than zero are considered as significantly, differentially expressed.

### Odorant receptivity changes

Relative differences in odorant receptivity between the *An. gambiae* and *An. quadriannulatus* were calculated from physiologic, odorant-response data from previously published functional deorphanization of *An. gambiae* odorant receptors [25,26]. The SSR data was first filtered to remove any *Ors* or chemicals that failed to elicit a 100 spikes/second increase over baseline in at least one assay. One hundred spikes per second was chosen to retain only more-robustly responding receptors and ligands in an attempt to mitigate any small potency differences that might exist between the species. Odor-induced decreases in spiking frequency were treated as indeterminate and treated as zero. The response of each *AgOr* (spikes/second increase) to each odorant was then weighted by the normalized abundance level (RPKM [83]) of that *Or*. Odorant responses in weighted-spikes-per-second were then summed for each odorant in each species, resulting in an “antennal receptivity” for that species. Finally, the interspecific “receptivity change” of the antenna was calculated by dividing the “antennal receptivity” of *An. gambiae* by that of *An. quadriannulatus*.

## Competing interests

The authors declare that they have no competing interests.

## Authors’ contributions

DCR, XZ, RJP, AR and LJZ discussed the experimental design and results. DCR and RJP preformed mosquito rearing, tissue collections and total RNA extractions. DCR and XZ preformed all bioinformatics analyses upon the RNAseq data sets. The AGC consortium generated the *An. quadriannulatus* reference genome assembly and authorized its use in this analysis in advance of the genome publication. DCR and XZ drafted the manuscript with subsequent contributions and revisions from DCR, XZ, RJP, AR and LJZ. All authors read and approved the final manuscript.

## Supplementary Material

Additional file 1 **A text file includes maximum-likelihood trees of the *OR*, *GR*, *IR*, and *OBP *genes in *An. gambiae *and *An. quadriannulatus.***
Click here for file

Additional file 2 A table listing the evolutionary rates for each orthologous group of chemosensory genes. Click here for file

Additional file 3 **A figure showing the correlation between protein distance and dN/dS ratio in each of the *OR*, *GR*, *IR*, and *OBP *families. Figure S1**. The two measurements of evolutionary rate are positively correlated in all chemosensory gene families. Scatter plots of protein distance and dN/dS ratio for orthologous gene pairs in each of the *OR* (A), *GR* (B), *IR* (C), and *OBP* (D) families. Spearman’s correlation (*rho*) between protein distances and dN/dS ratios are shown for each family. Click here for file

Additional file 4 **A figure showing the distribution of radical and conservative amino acid substitutions on the predicted OR topology. Figure S2.** Distribution of radical and conservative amino acid changes on predicted topological regions of *OR* genes. (A) Color coded representation of radical amino acid changes for each predicted topological regions of *ORs*. (B) Combined amino acid change per site for predicted transmembrane, intracellular, and extracellular regions. All values are averaged over all *OR* genes. Click here for file

Additional file 5 **A table listing the number of radical and conservative amino acid substitutions in each of the predicted topological regions of each orthologous group of *OR *genes.** Click here for file

Additional file 6 Reports the transcript abundances (in RPKMs) for every annotated AGAP in both species along with the “reliable log2” fold change (GFOLD value) for differences in abundance between species. Click here for file

Additional file 7 A table listing the statistical comparisons of median and variance values of transcript enrichment between genes in different quartiles of dN/dS ratios. Click here for file

Additional file 8 Reports the calculated odorant receptivity data to select AgOrs. Click here for file

Additional file 9 **A figure showing the high reproducibility of RNA-seq results between biologically replicated antennal samples. Figure S3.** Correlation of RNAseq data between biologically replicated antennal samples. Scatter plot of the numbers of reads uniquely mapping to more than 13,000 individual *An. gambiae* genes (AGAPs) in each of two RNAseq samples. For each sample, antennal tissue was resected from the same cohort of non-blood fed *An. gambiae* females and was taken one day apart at identical, light–dark time points (ZT6). Approximately 800 individual antennae comprise each sample. Spearman’s correlation (*rho*) between the two samples is shown. Click here for file

## The AGC Consortium

Daniel E. Neafsey, Aaron M. Berlin, George K. Christophides, Frank H. Collins, Scott J. Emrich, Michael C. Fontaine, William Gelbart, Matthew W. Hahn, Paul I. Howell, Fotis C. Kafatos, Daniel Lawson, Marc A. T. Muskavitch, Sarah Young, Robert Waterhouse and Nora J. Besansky.
