# Supplementary figures and images for: Antennal transcriptome profiles of anopheline mosquitoes reveal human host olfactory specialization in Anopheles gambiae
Source: BMC Genomics. 2013 Nov 1;14:749. doi: 10.1186/1471-2164-14-749 (PMC3833343; doi:10.1186/1471-2164-14-749)

**A***OR*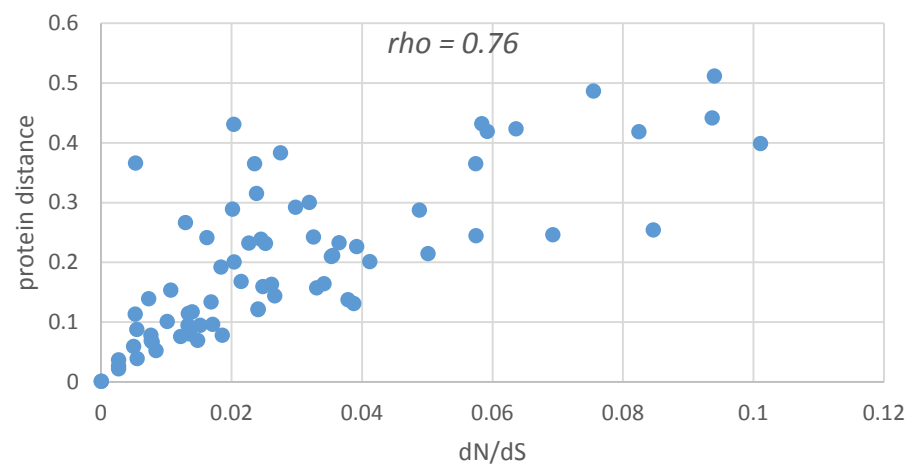**B***GR*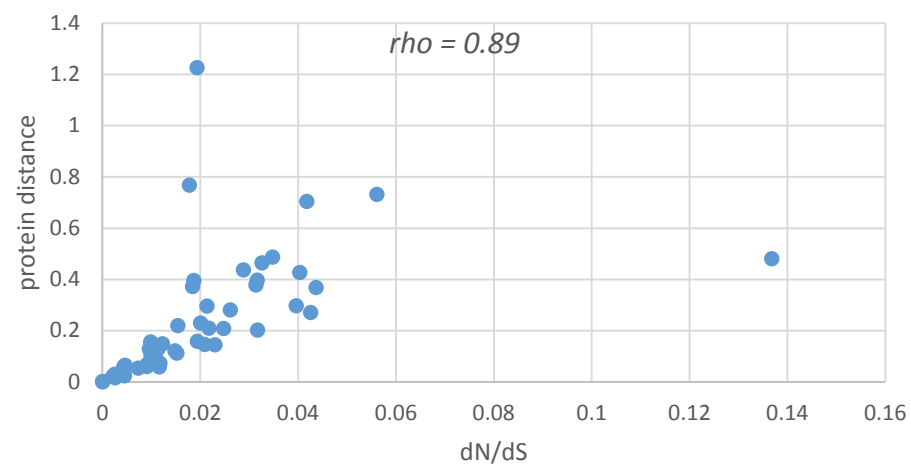**C***IR*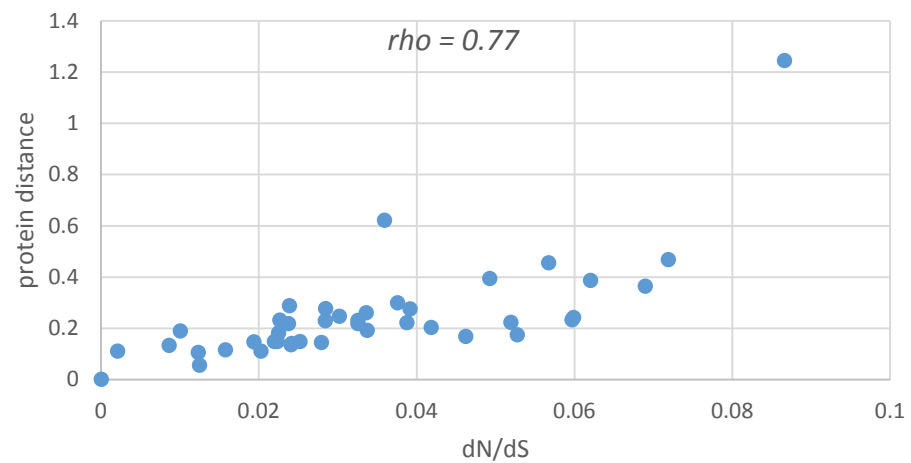**D***OBP*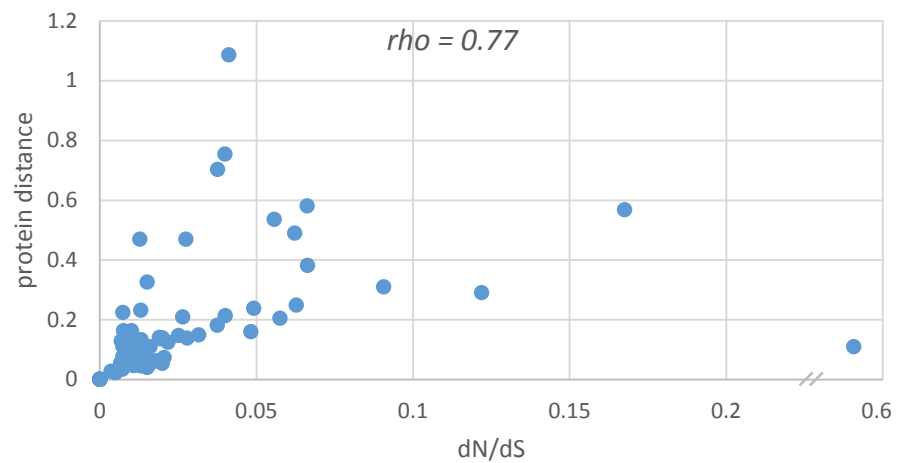

Supplement: Additional file 3 — A figure showing the correlation between protein distance and dN/dS ratio in each of the OR, GR, IR, and OBP families. Figure S1. The two measurements of evolutionary rate are positively correlated in all chemosensory gene families. Scatter plots of protein distance and dN/dS ratio for orthologous gene pairs in each of the OR (A), GR (B), IR (C), and OBP (D) families. Spearman’s correlation (rho) between protein distances and dN/dS ratios are shown for each family. [file 1471-2164-14-749-S3.pdf]

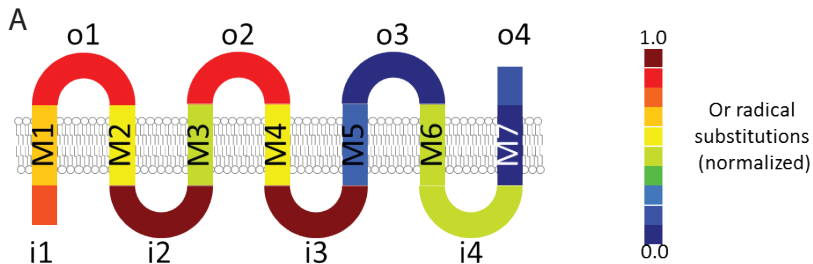

**B**

Average amino acid change per site

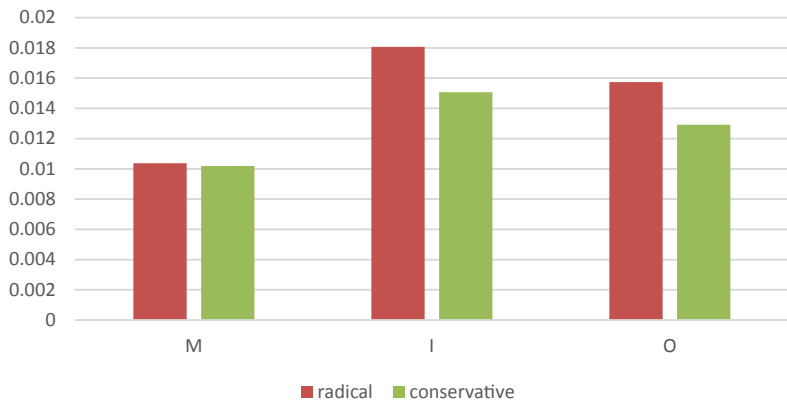

Supplement: Additional file 4 — A figure showing the distribution of radical and conservative amino acid substitutions on the predicted OR topology. Figure S2. Distribution of radical and conservative amino acid changes on predicted topological regions of OR genes. (A) Color coded representation of radical amino acid changes for each predicted topological regions of ORs. (B) Combined amino acid change per site for predicted transmembrane, intracellular, and extracellular regions. All values are averaged over all OR genes. [file 1471-2164-14-749-S4.pdf]

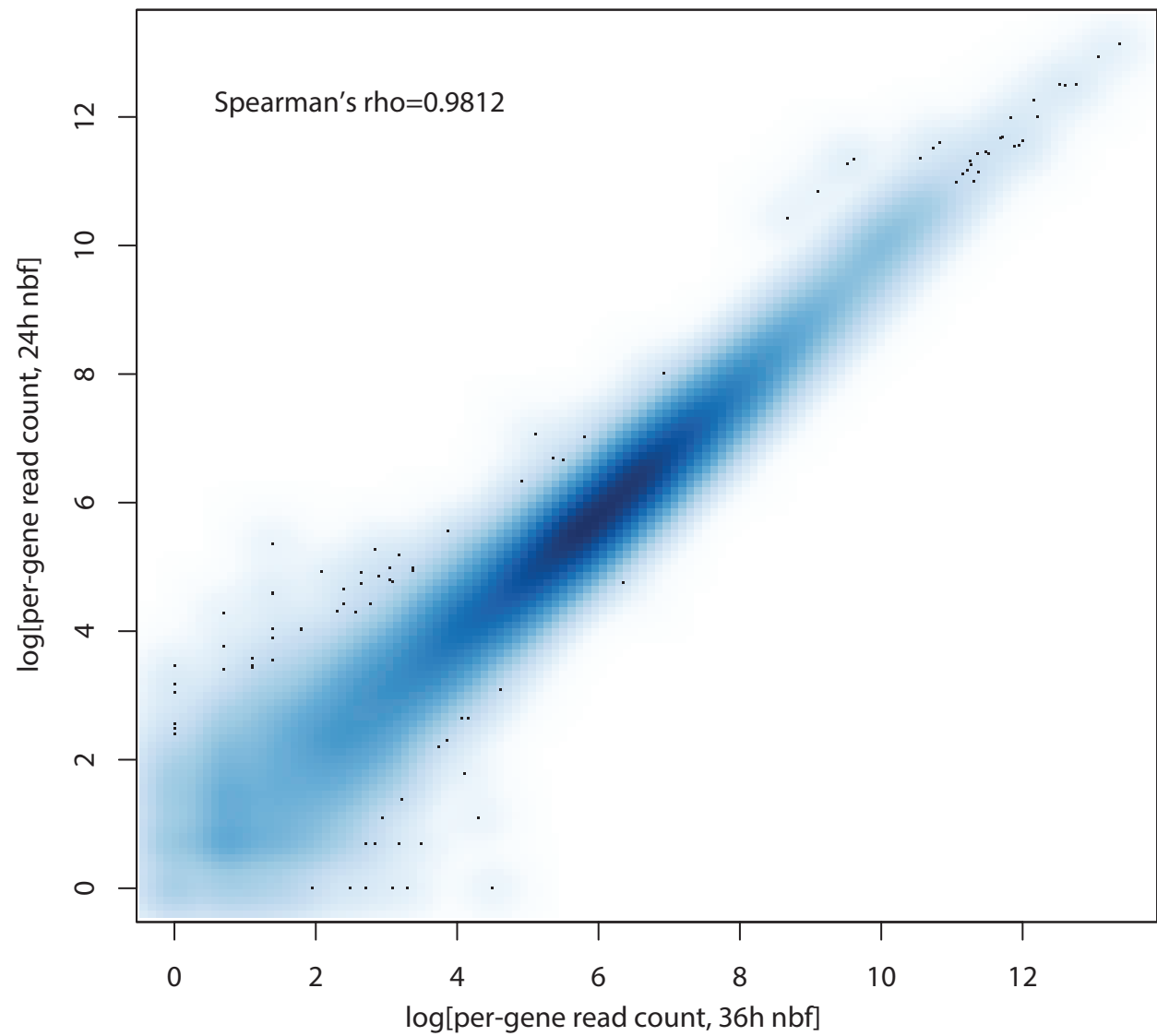

Supplement: Additional file 9 — A figure showing the high reproducibility of RNA-seq results between biologically replicated antennal samples. Figure S3. Correlation of RNAseq data between biologically replicated antennal samples. Scatter plot of the numbers of reads uniquely mapping to more than 13,000 individual An. gambiae genes (AGAPs) in each of two RNAseq samples. For each sample, antennal tissue was resected from the same cohort of non-blood fed An. gambiae females and was taken one day apart at identical, light–dark time points (ZT6). Approximately 800 individual antennae comprise each sample. Spearman’s correlation (rho) between the two samples is shown. [file 1471-2164-14-749-S9.pdf]
