# Supplementary material for: Antennal transcriptome profiles of anopheline mosquitoes reveal human host olfactory specialization in Anopheles gambiae
Source: BMC Genomics. 2013 Nov 1;14:749. doi: 10.1186/1471-2164-14-749 (PMC3833343; doi:10.1186/1471-2164-14-749)
Supplement: Additional file 7 — A table listing the statistical comparisons of median and variance values of transcript enrichment between genes in different quartiles of dN/dS ratios. [file 1471-2164-14-749-S7.docx]

**Table S4:**

| 1. **Median and variance of transcript enrichment for each quartile of dN/dS ratios:**   ***Or* genes** | | |
| --- | --- | --- |
|  | Median of absolute transcript enrichment | Variance of transcript enrichment |
| 1^st^ quartile | 0.0840588 | 0.2117066 |
| 2^nd^ quartile | 0.8885695 | 1.268659 |
| 3^rd^ quartile | 0.736114 | 2.871037 |
| 4^th^ quartile | 0.529047 | 1.777718 |
| 2^nd^+3^rd^+4^th^ quartiles | 0.848035 | 1.868296 |

| 1. **Comparisons of median and variance of transcript enrichment between quartiles:**   ***Or* genes** | | |
| --- | --- | --- |
|  | Median of absolute transcript enrichment* | Variance of transcript enrichment** |
| 1^st^ vs. 2^nd^ quartile | 0.002488 | 0.003628 |
| 1^st^ vs. 3^rd^ quartile | 0.08397 | 0.02898 |
| 1^st^ vs. 4^th^ quartile | 0.04925 | 0.02928 |
| 1^st^ vs. 2^nd^+3^rd^+4^th^ quartile | 0.00559 | 0.009608 |

* *p*-values of the Wilcoxon rank sum test of equality of median

** *p*-values of the Brown-Forsythe test of equality of variance

| 1. **Median and variance of transcript enrichment for each quartile of dN/dS ratios: transcriptome background** | | |
| --- | --- | --- |
|  | Median of absolute transcript enrichment | Variance of transcript enrichment |
| 1^st^ quartile | 0.503318 | 0.748001 |
| 2^nd^ quartile | 0.404235 | 0.6303521 |
| 3^rd^ quartile | 0.379404 | 0.6302595 |
| 4^th^ quartile | 0.373417 | 0.7727998 |
| 2^nd^+3^rd^+4^th^ quartiles | 0.387197 | 0.6878089 |

| 1. **Comparisons of median and variance of transcript enrichment between quartiles: transcriptome background** | | |
| --- | --- | --- |
|  | Median of absolute transcript enrichment* | Variance of transcript enrichment** |
| 1^st^ vs. 2^nd^ quartile | 0.000326 | 0.0003031 |
| 1^st^ vs. 3^rd^ quartile | 3.246e-07 | 5.188e-06 |
| 1^st^ vs. 4^th^ quartile | 4.552e-06 | 0.005662 |
| 1^st^ vs. 2^nd^+3^rd^+4^th^ quartile | 2.788e-08 | 7.204e-06 |

* *p*-values of the Wilcoxon rank sum test of equality of median

** *p*-values of the Brown-Forsythe test of equality of variance
